# Rhein antagonizes P2X_7_ receptor in rat peritoneal macrophages

**DOI:** 10.1038/srep14012

**Published:** 2015-09-10

**Authors:** Fen Hu, Fulin Xing, Ge Zhu, Guangxue Xu, Cunbo Li, Junle Qu, Imshik Lee, Leiting Pan

**Affiliations:** 1 The Key Laboratory of Weak-Light Nonlinear Photonics of Education Ministry, School of Physics and TEDA Applied Physics Institute, Nankai University, Tianjin, China; 2 School of Life Sciences, Lanzhou University, Lanzhou, China; 3 Shenzhen Key Laboratory of Micro-Nano Measuring and Imaging in Biomedical Optics, College of Optoelectronic Engineering, Shenzhen University, Shenzhen, China; 4 State Key Laboratory of Medicinal Chemical Biology, Nankai University, Tianjin, China

## Abstract

P2X_7_ receptor plays important roles in inflammation and immunity, and thereby it serves as a potential therapeutic target for inflammatory diseases. Rhein, an anthraquinone derivative, exhibits significant anti-inflammatory and immunosuppressive activities in therapy. However, the underlying mechanisms are largely unclear. Here, we aimed to investigate the effects of rhein on P2X_7_ receptor-mediated responses *in vitro.* In HEK293 cells expressing rat P2X_7_ receptor, we first found that rhein concentration-dependently blocked ATP-induced cytosolic calcium concentration ([Ca^2+^]_c_) elevation and pore formation of the plasma membrane, two hallmarks of the P2X_7_ receptor activation. These two inhibitory effects of rhein were also observed in rat peritoneal macrophages. Furthermore, rhein counteracted macrophage phagocytosis attenuation and suppressed reactive oxygen species (ROS) production triggered by ATP/BzATP. Meanwhile, rhein reduced ATP/BzATP-induced IL-1β release in lipopolysaccharide-activated macrophages. Prolonged application of ATP caused macrophage apoptosis, while the presence of rhein suppressed this cell cytotoxicity. Such ATP/BzATP-induced cellular reactions were also inhibited by a well-known rat P2X_7_ receptor antagonist, brilliant blue G, in a similar way to rhein. Together, our results demonstrate that rhein inhibit ATP/BzATP-induced [Ca^2+^]_c_ increase, pore formation, ROS production, phagocytosis attenuation, IL-1β release and cell apoptosis by antagonizing the P2X_7_ receptor in rat peritoneal macrophages.

Accumulating evidences indicated that injury and inflammatory processes were associated with increased concentration of extracellular ATP^1^,^2^, an important signaling molecule that could regulate numerous biological functions through interacting with purinergic P2 receptors at the cell surface^3^. The P2 receptors have two structurally and functionally distinct families: ligand-gated ion channel P2X receptors and G-protein coupled P2Y receptors^4^. P2X_7_ receptor, a member of the P2X receptor family^5^, is widely expressed in immune cells including monocytes^6^, macrophages^7^, mast cells^8^ and microglia^9^. The P2X_7_ receptor should be activated by the physiological agonist ATP at submillimolar or millimolar concentrations, which are 10–100 times higher than that of other P2X receptors activation^10^. P2X_7_ receptor can also be activated by 2′,3′-O-(benzoyl-4-benzoyl)-ATP (BzATP), an ATP analog, with a potency significantly greater than that of ATP, whereas the other P2X receptors are either insensitive or exhibit the opposite order of potency to BzATP^11^. Brief stimulation of the P2X_7_ receptor, like the other P2X receptors, opens a Ca^2+^-permeable channel, further resulting in an increase in cytosolic Ca^2+^ concentration ([Ca^2+^]_c_) through extracellular Ca^2+^ entry^12^. Prolonged activation of P2X_7_ receptor is unique in triggering the formation of large nonselective membrane pores which are permeable to hydrophilic molecules with molecular mass ≤900 Da^13^,^14^. This membrane permeabilization was suggested to cause disruption of ionic gradients and/or efflux of vital intracellular molecules, mitochondrial dysfunctioning, DNA fragmentation, and eventually cell death^14^,^15^. Indeed, P2X_7_ was proposed to be a critical player for ATP-triggered cell death by necrosis and/or apoptosis in many cell types such as mast cells^16^, thymocytes^17^, lymphocytes^18^, astrocytes^19^ and macrophages^20^.

Recently, more and more evidences showed a crucial role of P2X_7_ receptor in immune responses and inflammation^21^,^22^,^23^, as well as in the pathogenesis of inflammatory diseases including arthritis^24^, chronic inflammatory pain and neuroinflammation^25^. These studies supported to the notion that P2X_7_ receptor exhibited salient pharmacological and functional properties in inflammation and immunity^23^. Macrophages, a type of multifunctional immune cells, were demonstrated to express P2X_7_ receptor abundantly^7^. P2X_7_ receptor was also reported to be involved in mediating inflammatory responses of macrophages such as chemotaxis, oxygen radical generation, caspases activation, cytokine secretion and phagocytosis^26^,^27^. For instance, ATP was able to promote massive release of mature pro-inflammatory cytokine interleukin-1β (IL-1β) via P2X_7_ receptor activation in bacterial lipopolysaccharide (LPS)-primed macrophages^26^,^28^. Furthermore, P2X_7_ receptor contributed to ATP-triggered reactive oxygen species (ROS) generation in macrophages^27^,^29^ as well as other P2X_7_ expressing immune cells^13^,^30^. Besides, short-term exposure to millimolar extracellular ATP significantly attenuated phagocytosis of macrophages^31^ and microglia^32^ by acting on P2X_7_ receptors.

Rhein (4, 5-dihydroxyanthraquinone-2-carboxylic acid) is a constituent of the rhizome of rhubarb, a traditional Chinese herb that is used as a laxative and stomachic drug. Clinical studies and experiments with animal disease models or different functional cells demonstrated that rhein exerted multiple functions including anti-carcinogenesis^33^,^34^, antioxidant^35^, anti-inflammation and immunosuppression^36^,^37^,^38^. For instance, rhein is an active metabolite of osteoarthritis drug diacetylrhein, which could alleviate inflammatory osteoarthritis^39^. As mentioned above, the P2X_7_ receptor is a potential therapeutic target for inflammatory diseases. There also have been enormous efforts in developing novel drug-like P2X_7_ receptor antagonists such as brilliant blue G (BBG)^40^, KN-62^41^, A740003^42^ and AACBA^43^. However, relevant clinical application is still restricted due to lacking of natural potent antagonists. In this study, we found that rhein could inhibit the P2X_7_ receptor-mediated rat peritoneal macrophages responses such as [Ca^2+^]_c_ increases, pore formation, ROS production, IL-1β release, phagocytosis attenuation and cell apoptosis. Our results suggested that rhein had significant inhibitory effects on these responses as a potential antagonist of P2X_7_ receptor.

## Results

### Effects of Rhein on ATP-induced [Ca^2+^]_c_ increase and pore formation in rP2X_7_-HEK293 cells

**Figure 1:**
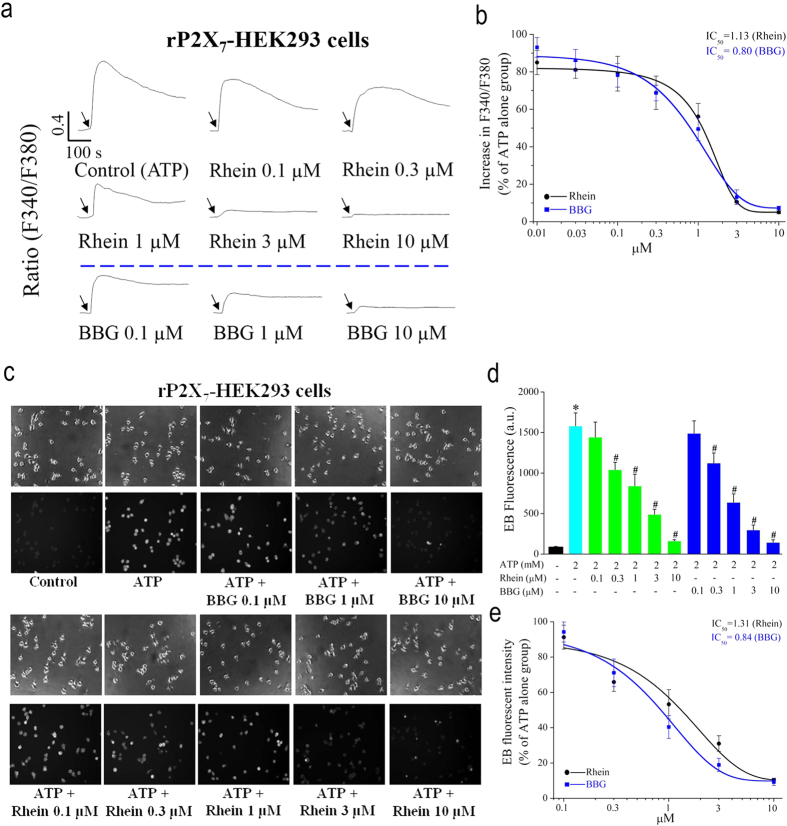
Rhein blocked ATP-induced [Ca^2+^]_c_ increase and pore formation in rP2X7- HEK293 cells in a concentration-dependent manner. (**a**) Representative tracings of [Ca^2+^]_c_ changes for cells treated with ATP (5 mM) alone and cells preincubated with different concentrations of rhein (0.1, 0.3, 1, 3, 10 μM) or BBG (0.1, 1, 10 μM). Arrows indicated applications of ATP. (**b**) The statistic peak values of increase in F340/F380 ratio after application of ATP (5 mM) alone and in the presence of rhein (0.01, 0.03, 0.1, 0.3, 1, 3, 10 μM) or BBG (0.01, 0.03, 0.1, 0.3, 1, 3, 10 μM) (*n* = 20 cells for each case). The smooth curve represents the fit with the Boltzmann equation. (**c**) Representative phase contrast images visualizing all cells (upper panel) and ethidium bromide (EB) fluorescent images showing dye uptake positive cells (lower panel). (**d**) Summary of the fluorescence intensity measured in each experimental group (*n* = 60 cells for each case). *P < 0.05, compared to control group (cells treated with vehicle solution: no ATP, BBG or rhein); ^#^*P* < 0.05, compared to ATP alone group. (**e**) The statistic valu**e**s of the relative EB uptake rates in rhein and BBG group (ATP alone group was taken as 100%). The smooth curve represents the fit with the Boltzmann equation.

To examine whether rhein inhibits the P2X_7_ receptor *in vitro*, we first tested the effects of rhein on ATP-induced [Ca^2+^]_c_ increase and pore formation, two hallmarks of P2X_7_ receptor activation, in human embryonic kidney 293 cells stably expressing rat P2X_7_ receptor (rP2X_7_-HEK293 cells). Firstly, application of ATP (5 mM) evoked a rapid increase in [Ca^2+^]_c_ followed by a sustained high plateau in rP2X_7_-HEK293 cells (Fig. 1a). Then, ATP-evoked [Ca^2+^]_c_ increases were significantly reduced in a dose dependent manner by pretreatment with rhein at 0.01, 0.03, 0.1, 0.3, 1, 3, 10 μM, respectively. Fitting the mean data with the Boltzmann equation yielded an IC_50_ of 1.13 μM (Fig. 1b). In addition, BBG, a potent rat P2X_7_ receptor antagonist^40^, was used as a positive control to determine the potency and specificity of rhein. As shown in Fig. 1a (third line), the ATP-triggered [Ca^2+^]_c_ increase was also remarkably inhibited by BBG (Representative traces of 0.1, 1, 10 μM were shown). The corresponding IC_50_ of BBG is 0.80 μM (Fig. 1b). Another salient functional property of P2X_7_ receptor is that prolonged activation of the receptor causes formation of nonselective membrane pores which enables the cells to uptake cationic fluorescent dyes such as ethidium bromide (EB). Therefore, we subsequently examined the effect of rhein on membrane pore formation by detecting the ATP-induced uptake of EB in rP2X_7_-HEK293 cells. It showed that EB uptake triggered by ATP (2 mM) was concentration-dependently inhibited by rhein with an IC_50_ of 1.31 μM (Fig. 1c–e). In addition, ATP-triggered EB uptake was remarkably inhibited by BBG with an IC_50_ of 0.84 μM (Fig. 1c–e), indicating the involvement of P2X_7_ receptor. Taken together, these data indicated that rhein had efficient inhibitory effects on ATP-induced [Ca^2+^]_c_ increase and pore formation by antagonizing P2X_7_ receptor in rP2X_7_-HEK293 cells.

### Effects of Rhein on ATP/BzATP-induced [Ca^2+^]_c_ increase in rat peritoneal macrophages

**Figure 2:**
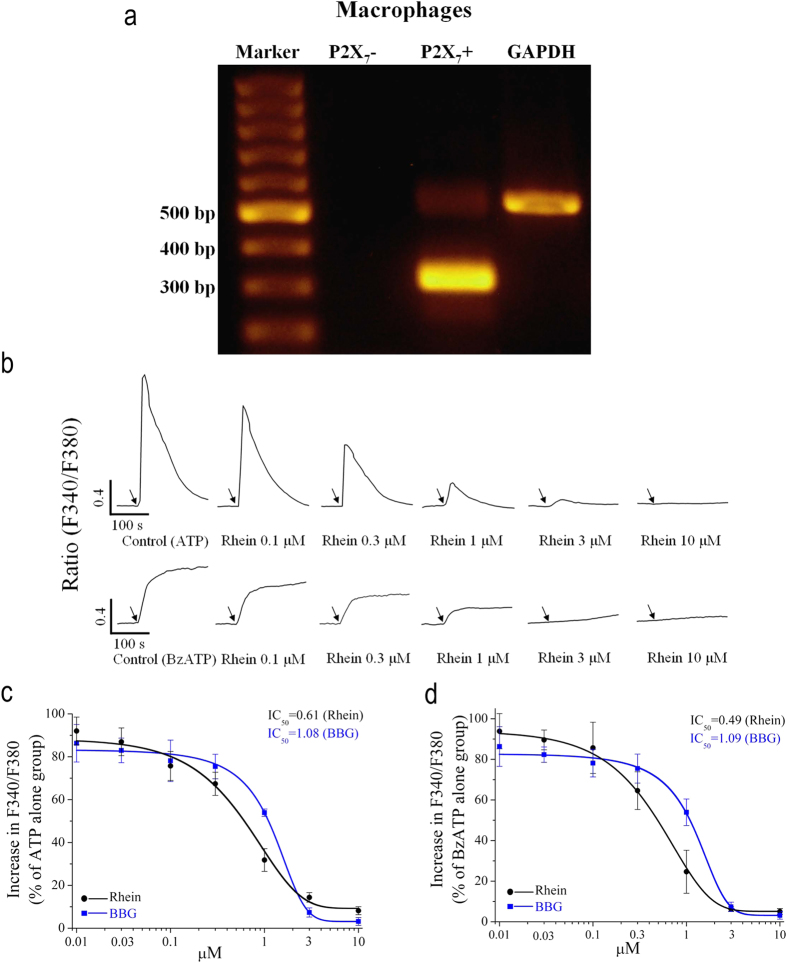
Rhein inhibited the increases in [Ca^2+^]_c_ evoked by ATP/BzATP in rat peritoneal macrophages. (**a**) P2X_7_ mRNA was detected in rat peritoneal macrophages by RT-PCR. The four lanes in the gel were as follows: Marker (with a list of standardized DNA sequences from 100 bp to 1000 bp); P2X_7_+ (experimental group with primers directed towards the P2X_7_ mRNA); P2X_7_- (negative control group with nuclease-free water instead of DNA template); GAPDH (positive control group with primers directed towards the GAPDH mRNA). (**b**) Representative [Ca^2+^]_c_ traces for stimulating macrophages with ATP (5 mM) or BzATP (500 μM) after pretreatment with rhein (0.1, 0.3, 1, 3, 10 μM). Rhein was added for 20 min before the addition of ATP. Arrows indicated the application of ATP or BzATP. C/D: The statistic peak values of increase in F340/F380 ratio after application of ATP (**c**) or BzATP (**d**) alone and in the presence of rhein (0.01, 0.03, 0.1, 0.3, 1, 3, 10 μM) or BBG (0.01, 0.03, 0.1, 0.3, 1, 3, 10 μM) (*n* = 20 cells for each case). The smooth curve represents the fit with the Boltzmann equation.

Similarly, we monitored the effects of rhein on ATP/BzATP-induced [Ca^2+^]_c_ increase in rat peritoneal macrophages. It has been well established that P2X_7_ receptor is highly expressed in macrophages^7^. In this study, we also confirmed the presence of P2X_7_ receptors in rat peritoneal macrophages using RT-PCR (Fig. 2a). As expected, ATP (5 mM) elicited a rapid [Ca^2+^]_c_ increase in macrophages (Fig. 2b). Meanwhile, rhein robustly inhibited this [Ca^2+^]_c_ increase (Fig. 2b). The inhibition was dependent on the concentration of rhein with an IC_50_ of 0.61 μM (Fig. 2c). Furthermore, BzATP (500 μM), a specific agonist of P2X_7_ receptor, induced strong sustained elevation of [Ca^2+^]_c_ in macrophages as reported by many previous studies. Rhein also concentration-dependently inhibited BzATP-induced increase in [Ca^2+^]_c_, with an IC_50_ of 0.49 μM (Fig. 2d). Besides, BBG exhibited similar inhibitory effects on ATP- and BzATP-induced increases in [Ca^2+^]_c_ with IC_50_ of 1.08 μM and 1.09 μM, respectively (Fig. 2c,d). These results together suggested an efficient inhibitory effect of rhein on [Ca^2+^]_c_ increase mediated by P2X_7_ receptor activation in macrophages.

### Effect of Rhein on ATP-induced pore formation in rat peritoneal macrophages

**Figure 3:**
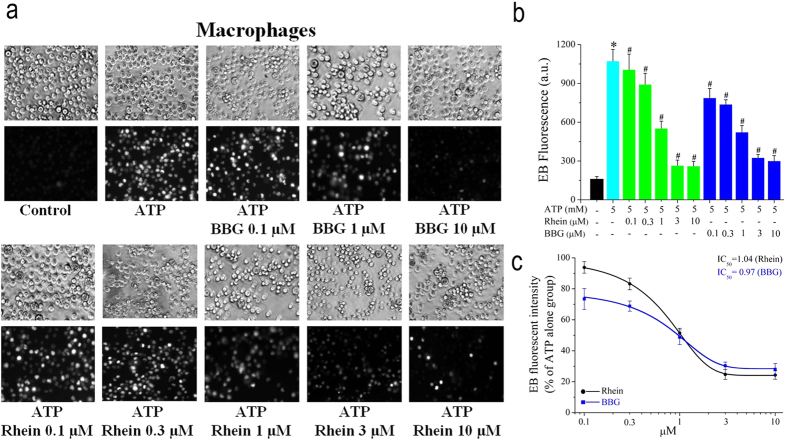
Rhein suppressed pore formation resulted from ATP in rat peritoneal macrophages. (**a**) Representative phase contrast images visualizing all cells (upper panel) and ethidium bromide (EB) fluorescent images showing dye uptake positive cells (lower panel). (**b**) Summary of the EB fluorescence intensity measured in each experimental group (*n* = 150 cells for each case). *P < 0.05, compared to control group (macrophages treated with vehicle solution: no ATP, BBG or rhein). ^#^*P* < 0.05, compared to ATP alone group. (**c**) The statistic values of the relative EB uptake rates in rhein and BBG group (ATP alone group was taken as 100%). The smooth curve represents the fit with the Boltzmann equation. ^#^*P* < 0.05, compared to ATP alone group.

We further examined whether rhein inhibited pore formation by detecting ATP-induced uptake of EB in macrophages. As shown in Fig. 3, the EB uptake of macrophages induced by ATP was also concentration-dependently inhibited by rhein with an IC_50_ of 1.04 μM. The mean fluorescent intensity was significantly reduced to 94 ± 4% (ATP + 0.1 μM rhein group), 83 ± 4% (ATP + 0.3 μM rhein group), 51 ± 3% (ATP + 1 μM rhein group), 25 ± 3% (ATP + 3 μM rhein group) and 24 ± 3% (ATP + 10 μM rhein group) as compared with the ATP alone group (Fig. 3c). ATP-induced EB uptake was also suppressed by BBG in a similar way to rhein, with an IC_50_ of 0.97 μM (Fig. 3c). These results suggested that rhein could significantly block the pore formation due to ATP-induced P2X_7_ receptors activation in macrophages.

### Effects of Rhein on ATP/BzATP-induced phagocytosis attenuation in rat peritoneal macrophages

**Figure 4:**
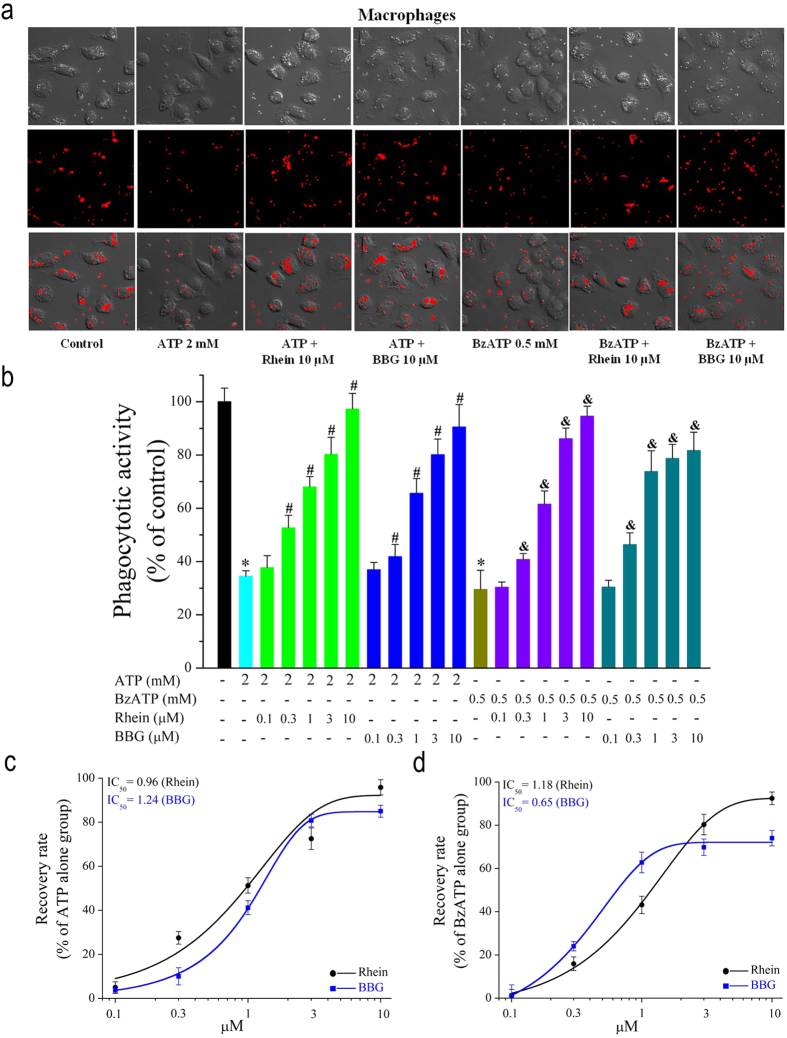
Rhein prevented ATP/BzATP-induced phagocytosis decrease in rat peritoneal macrophages. (**a**) Representative bright-field (upper panel), nile red fluorescent images (middle panel) and overlay images (lower panel) showing the effects of ATP/BzATP alone or together with rhein/BBG on the uptake of fluorescent particles (red color). (**b**) Summary of the relative phagocytic activity in each group (Control group was taken as 100%). Macrophages were pretreated with rhein (0.1, 0.3, 1, 3, 10 μM) or BBG (0.1, 0.3, 1, 3, 10 μM) for 3 h, and then applied with ATP (2 mM) or BzATP (500 μM) and fluorospheres (10 μl) for additional 45 min. All data are presented as means ± SD (*n* = 100 cells for each case) *P < 0.05, compared to co*n*trol group; ^#^*P* < 0.05, compared to ATP alone group; ^&^*P* < 0.05, compared to BzAT*P* alone group. (**c**) Statistic data of the recovery rates of phagocytic activity of ATP group. The recovery rates can be derived from the values of shown in (**b**). The smooth curve represents the fit with the Boltzmann equation with an EC_50_ of 0.96 μM (rhein) and 1.24 μM (BBG). (**d**) Statistic data of the recovery rates of phagocytic activity of BzATP group. The smooth curve represents the fit with the Boltzmann equation with an EC_50_ of 1.18 μM (rhein) and 0.65 μM (BBG).

Phagocytosis is a specific form of endocytosis which takes relatively large particles (>250 nm) into vacuoles. ATP is reported to attenuate phagocytosis of macrophages^31^ by acting on P2X_7_ receptors. Therefore, the effect of rhein on ATP/BzATP-evoked phagocytosis attenuation of macrophages was examined. The phagocytic activity of macrophages was assessed by the uptake of 1.0 μm diameter nile red fluorescent carboxylate-modified microspheres (Fig. 4). As shown in Fig. 4a, compared to control group, stimulation with ATP (2 mM) or BzATP (500 μM) for 45 min evoked an obvious decrease in phagocytosis of microspheres, indicating that exposure to ATP or BzATP could lead to phagocytosis attenuation of macrophages. However, pretreatment with rhein (0.1, 0.3, 1, 3, 10 μM) inhibited the decrease in microspheres uptake in a concentration-dependent manner (Fig. 4a,b). BBG (0.1, 0.3, 1, 3, 10 μM) also counteracted this negative effect of ATP on the phagocytosis of macrophages, supporting that P2X_7_ receptor contributed to the phagocytosis attenuation induced by ATP. Moreover, rhein inhibited BzATP-induced macrophage phagocytosis attenuation (Fig. 4a,b). The influences of rhein or BBG on phagocytic activity of macrophages were expressed as recovery rate, showed in Fig. 4c,d. It was found that pretreatment with 0.3, 1, 3 and 10 μM rhein increased the recovery percentage of ATP to 27.5%, 51.3%, 72.5%, and 95.8%, respectively. Pretreatment with 0.3, 1, 3 and 10 μM BBG increased the recovery rate of ATP to 10%, 41.2%, 80.8% and 85%, respectively. In addition, pretreatment with 0.3, 1, 3 and 10 μM rhein increased the recovery percentage of BzATP to 15.9%, 43.2%, 80.3% and 92.5%, respectively. The IC_50_ value of rhein and BBG on ATP-induced phagocytosis attenuation was 0.99 μM and 1.24 μM, respectively (Fig. 4c). The IC_50_ value of rhein and BBG on BzATP-induced phagocytosis attenuation was 1.18 μM and 0.65 μM, respectively (Fig. 4d). These data together indicated that rhein could block the influence of ATP/BzATP on the phagocytic activity, which is exerted through activating the P2X_7_ receptors.

### Effects of Rhein on ATP/BzATP-evoked ROS production in rat peritoneal macrophages

**Figure 5:**
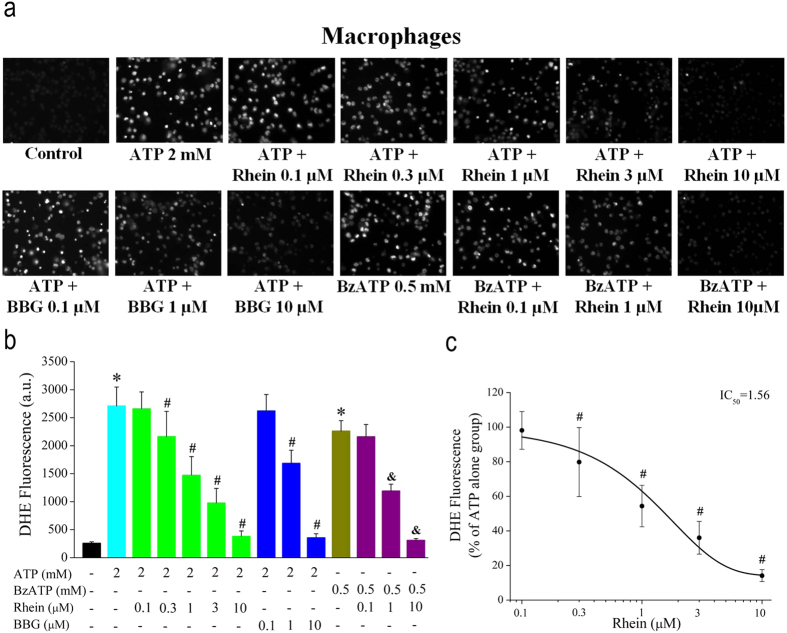
Rhein blocked ATP/BzATP-induced ROS production in rat peritoneal macrophages. (**a**) Thefluorescence intensity of dihydroethidium (DHE) represents the ROS concentration. The cells were pretreated with rhein (0.1, 0.3, 1, 3, 10 μM) or BBG (0.1, 1, 10 μM) for 3 h, then, stimulated with ATP (2 mM) or BzATP (0.5 mM) for additional 30 min. (**b**) Statistic results of the DHE fluorescence intensity from three independent experiments (*n* = 90 cells for each case). *P < 0.05, compared to control group; ^#^*P* < 0.05, compared to ATP alone group; ^&^*P* < 0.05, compared to BzATP alone group. (**c)** Summary of the relative percentage of fluorescence intensity in rhein group (ATP alone group was taken as 100%). The smooth curve represents the fit to the Boltzmann equation with an IC_50_ of 1.56 μM. *P < 0.05, compared to ATP alone group.

Next, the effect of rhein on ATP/BzATP-evoked intracellular ROS production was detected by fluorescent imaging with dihydroethidium (DHE). As shown in Fig. 5, compared with control (cells treated with vehicle solution), the stimulation with ATP (2 mM) or BzATP (0.5 mM) for 30 min induced remarkable generation of intracellular ROS. However, treatment with rhein (10 μM) or BBG (10 μM) alone had no obvious effect on ROS production (data not shown). Three hours’ pretreatment with rhein (0.1, 0.3, 1, 3, 10 μM) or BBG (0.1, 1, 10 μM) effectively suppressed the ROS production evoked by ATP in concentration-dependent manners, respectively. The IC_50_ of rhein was 1.56 μM (Fig. 5c). Rhein also blocked BzATP-induced ROS generation (Fig. 5a,b). Taken together, these results suggested a suppressive effect of rhein on ATP/BzATP-induced ROS production, which was mediated by the activation of P2X_7_ receptor.

### Effects of Rhein on ATP/BzATP-induced IL-1β release in LPS-activated macrophages

**Figure 6:**
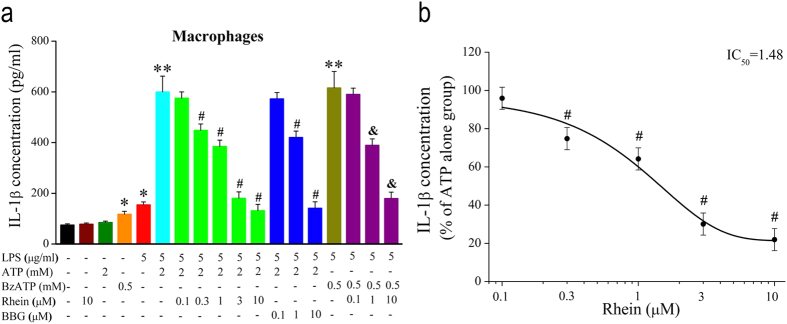
Rhein inhibited ATP/BzATP-induced IL-1β release in LPS-activated macrophages. (**a**) Macrophages were pretreated with LPS (5 μg/ml)and rhein (0.1, 0.3, 1, 3, 10 μM) or BBG (0.1, 1, 10 μM) for 5 h simultaneously, then stimulated with ATP (2 mM) or BzATP (0.5 mM) for additional 2 h. IL-1β production in supernatants was measured by ELISA kits. The concentration of IL-1β (pg/ml) in each group was determined by the standard curve. Data shown are mean ± SD (*n* = 5 for each group). *P < 0.05, compared to control group; **P < 0.05, compared to LPS-primed group; ^#^*P* < 0.05, compared to ATP + LPS group; ^&^*P* < 0.05, compared to BzATP + LPS group. (**b**) The statistic data of the relative percentage of IL-1β release in rhein group (ATP alone group was taken as 100%). The smooth curve represents the fit to the Boltzmann equation with an IC_50_ of 1.48 μM. ^#^*P* < 0.05, compared with ATP + LPS group.

Furthermore, we examined ATP/BzATP-induced IL-1β release from LPS-activated rat peritoneal macrophages, and further evaluated whether rhein inhibited the IL-1β secretion induced by ATP/BzATP. As previously reported^26^, the pro-IL-1β is accumulated in macrophages induced by bacterial LPS stimulation. Therefore, macrophages were pretreated with 5 μg/ml LPS, LPS + rhein and LPS + BBG for 5 h, respectively. Then, the cells were stimulated with ATP (2 mM) or BzATP (500 μM) for additional 2 h. As shown in Fig. 6a, both ATP and BzATP evoked massive secretion of IL-1β in LPS-primed macrophages (ATP group: 599.5 ± 62.4 pg/ml, 8 folds increase compared with the basal level 74.9 ± 4.5 pg/ml in control; BzATP group: 615.5 ± 65.0 pg/ml, 8.2 folds increase compared with control). Moreover, rhein reduced the IL-1β release triggered by ATP in a concentration-dependent way with an IC_50_ of 1.48 μM (Fig. 6b). Rhein also inhibited BzATP-induced IL-1β secretion. Similarly, BBG exhibited suppressive effects on IL-1β secretion. In addition, rhein alone had no effect on the basal IL-1β secretion (78.1 ± 5.1 pg/ml). ATP or LPS alone cannot cause massive extracellular accumulation of IL-1β (85.0 ± 5.0 pg/ml and 117.5 ± 11.1 pg/ml, respectively). Since ATP-induced IL-1β release from LPS-primed macrophages is directly associated with the activation of P2X_7_ receptor^26^,^28^, these results indicated that rhein reduced ATP/BzATP-induced IL-1β release by inhibiting the activation of P2X_7_ receptors.

### Effect of Rhein on ATP-induced death of rat peritoneal macrophages

**Figure 7:**
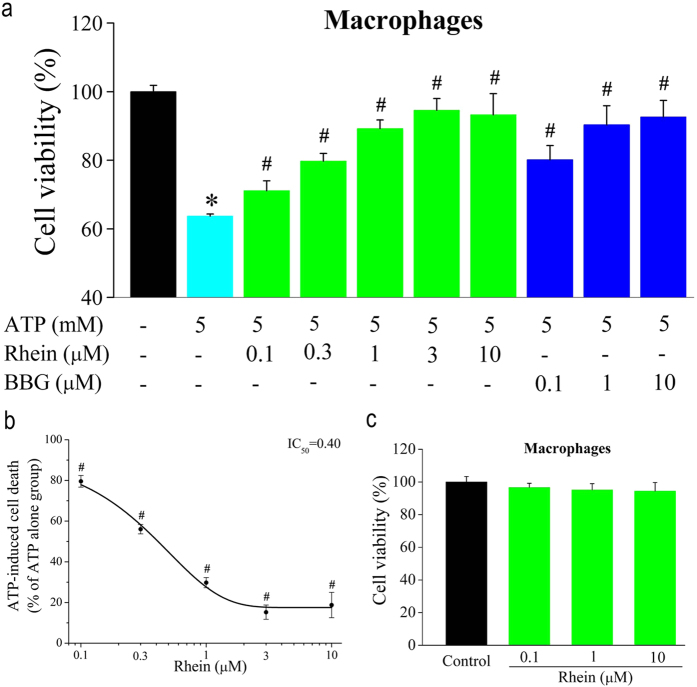
Rhein reduced ATP-induced macrophage cell death. (**a**) Cell viability in control (no ATP, BBG and rhein) and cells exposed to ATP (5 mM) in the presence of rhein (0.1, 0.3, 1, 3, 10 μM) and BBG (0.1, 1, 10 μM), respectively. Data represent the mean ± SD (*n* = 15 wells for each case). *P < 0.05 compared to control group; ^#^*P* < 0.05, compared to ATP alone group. (**b**) The statistic data of the percentage of dead cells in rhein group (ATP alone group was taken as 100%). The smooth curve represents the fit to the Boltzmann equation with an IC_50_ of 0.40 μM. *P < 0.05 compared to ATP alone group. (**c**) Cell survival in cells treated with rhein at 0.1, 1, 10 μM, respectively. There was no significant difference between treated cells and control (no rhein) (*n* = 15 wells for each case).

We tested whether rhein prevented macrophage death induced by high extracellular ATP. Stimulation with ATP (5 mM) evidently reduced macrophage viability as summarized in Fig. 7a. However, rhein reversed ATP-induced macrophage death in a concentration-dependent manner, with significant inhibition at concentrations of ≥0.1 μM. Fitting the mean data with the Boltzmann equation derived an IC_50_ of 0.4 μM (Fig. 7b). In contrast, there was no cytolytic action with concentrations up to 10 μM rhein (Fig. 7c). Besides, the ATP-induced cell death was suppressed by BBG in a similar fashion. These data suggested that rhein inhibited ATP-induced cell death through blocking P2X_7_ receptors in rat peritoneal macrophages.

### Effect of Rhein on ATP-induced mitochondrial membrane depolarization (MMP) and caspase-3/7 activation in rat peritoneal macrophages

**Figure 8:**
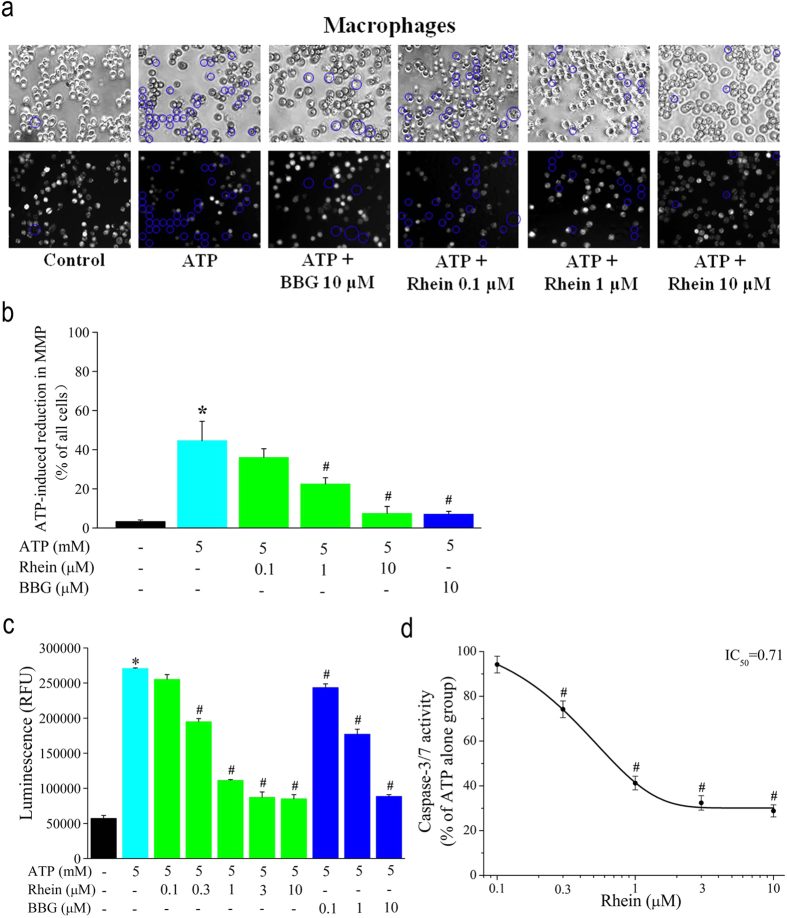
Rhein abolished ATP-induced mitochondrial membrane depolarization and caspase3/7 activation in rat peritoneal macrophages. (**a**) Macrophages were stained with Rhodamine 123 to assess MMP. A decrease in fluorescence intensity represents mitochondrial membrane depolarization. Representative phase contrast images visualizing all cells (upper panel) and Rhod**a**mine 123 fluorescent images differentiating normal and MMP depolarized/disrupted cells (lower panel). Cells were treated for 3 h with ATP (5 mM) alone or together with rhein (0.1, 1, 10 μM) or BBG (10 μM). Control cells were treated with neither ATP nor inhibitors. Cells marked by blue circles represented depolarization even disruption of the mitochondrial membrane potential. (**b**) Summary of the percentages of cells showing depolarized mitochondrial membrane obtained from three independent experiments (*n* = 50 cells for each case). *P < 0.05 compared to control group; ^#^*P* < 0.05, compared to ATP alone group (no BBG and rhein). (**c**) Caspase-Glo assay kit was used to measure caspase 3/7 activities in macrophages. Cells were treated with ATP (5 mM) alone or together with rhein (0.1, 0.3, 1, 3 and 10 μM) or BBG (0.1, 1 and 10 μM). Control cells were treated with neither ATP nor inhibitors. Data shown are mean ± SD of three independent experiments. *P < 0.05 compared to control group; ^#^*P* < 0.05, compared to ATP alone group. D: Summary of the percentage of caspase 3/7 activity in rhein group (ATP alone group was taken as 100%). The smooth curve represented the fit to the Boltzmann equation with an IC_50_ of 0.71 μM. *P < 0.05 compared to ATP alone group (*n* = 3 for each case).

To estimate whether apoptosis was involved in ATP-induced macrophage death, we observed the changes of MMP using rhodamine 123. Decrease in the fluorescence intensity of rhodamine 123 represents mitochondrial membrane depolarization. As shown in Fig. 8a, ATP (5 mM) induced remarkable depolarization even disruption of the inner mitochondrial transmembrane potential after stimulation for 3 h. In contrast, ATP-induced reduction of MMP was significantly reversed by pretreating the cells with rhein (1, 10 μM) or BBG (10 μM). As summarized in Fig. 8b, the percentages of cells showing depolarized/disrupted mitochondrion membrane potential were 3.3 ± 0.8% (control group), 44.6 ± 9.9% (ATP alone group), 36.1 ± 4.4% (ATP + 0.1 μM rhein group), 22.5 ± 3.1% (ATP + 1 μM rhein group), 7.5 ± 3.5% (ATP + 10 μM rhein group), and 7.1 ± 1.4% (ATP + 10 μM BBG group), respectively.

Simultaneously, we measured caspase 3/7 activity in macrophages to determine the apoptotic events. Stimulation with ATP for 3 h evoked an over five folds increase in the luminescence of caspase3/7 compared to the control. This increase in caspase activity was concentration-dependently inhibited by rhein (Fig. 8c) with an IC_50_ of 0.71 μM (Fig. 8d). The mean caspase activity was significantly reduced to 94.2 ± 3.8% (ATP + 0.1 μM rhein group), 74.2 ± 3.7% (ATP + 0.3 μM rhein group), 41.2 ± 3.0% (ATP + 1 μM rhein group), 32.4 ± 3.2% (ATP + 3 μM rhein group) and 28.8 ± 2.7% (ATP + 10 μM rhein group) compared to ATP alone group. In addition, BBG also exhibited similar inhibitory effects on ATP-evoked caspase 3/7 activation (Fig. 8c). These results indicated that rhein had a significant inhibitory effect on the ATP-induced apoptosis by antagonizing P2X_7_ receptor.

### Discussion

In the present work, we aimed to investigate the effects of rhein on rat P2X_7_ receptor by detecting the influences of rhein on various representative cellular responses or biochemical consequences mediated by P2X_7_ receptor activation. It has been established that millimolar concentrations of ATP are needed for P2X_7_ receptor activation^5^,^10^, and the optimal concentration of ATP chosen for each experiment in this study relies on previous reports^32^,^44^,^45^ and our preliminary experiments (Supplementary Fig. S1). Firstly, rP2X_7_-HEK293 cells, a cell line stably expressing the recombinant rat P2X_7_ receptor, were used to determine the effects of rhein on the P2X_7_ receptor through monitoring the change of [Ca^2+^]_c_ and pore formation, two hallmark properties directly associated with P2X_7_ receptor activation. Except P2X_7_ receptor, other P2X receptors do not exist in rP2X_7_-HEK293 cells. Thus, adoption of this cell line is a good approach to exclude the effects of other receptors and screen for specific antagonists for P2X_7_ receptor. Our results showed that ATP (5 mM) evoked an increase in [Ca^2+^]_c_ via activation of P2X_7_ receptor in rP2X_7_-HEK293 cells (Fig. 1a). Rhein had an efficient inhibition on this P2X_7_-mediated calcium response in a dose-dependent manner, which was similar to that of the specific P2X_7_ receptor blocker BBG (Fig. 1a,b). Furthermore, we found that rhein also potently blocked ATP-triggered typical large transmembrane pores formation in rP2X_7_-HEK293 cells (Fig. 1c,d). These data presented initial evidence that rhein may be a potential antagonist for rat P2X_7_ receptor. Based on the preliminary results acquired from rP2X_7_-HEK293 cells, the major focus of our study was to examine the effects of rhein on P2X_7_ receptor-mediated inflammatory responses in a functional immune cell type, rat peritoneal macrophages. Our results showed that stimulation of macrophages with P2X_7_ receptor agonists ATP/BzATP also induced rapid [Ca^2+^]_c_ increase (Fig. 2) and pore formation (Fig. 3), which was similar to those of rP2X_7_-HEK293 cells.

Subsequently, we investigated several other P2X_7_ receptor-mediated processes in rat peritoneal macrophages. As mentioned in *introduction*, P2X_7_ receptor is a key player in ATP-induced maturation and extracellular release of proinflammatory cytokine IL-1β^28^. Moreover, the activation of P2X_7_ receptor is necessary for the generation of ROS and reduction of phagocytic activity triggered by millimolar ATP in macrophages^27^,^29^,^31^. Here we showed that both ATP/BzATP strongly enhanced IL-1β release in LPS-activated macrophages (Fig. 6). And the experiment with BBG further illustrated that these consequences were mediated by P2X_7_ receptor activation (Fig. 6). Meanwhile, ATP/BzATP elicited remarkable intracellular ROS production (Fig. 5) and attenuated the phagocytic activity of macrophages (Fig. 4), which were also due to the activation of P2X_7_ receptor. These results were in good accordance with previous literatures with regard to P2X_7_ receptor expressing immune cells including macrophages^27^,^29^,^31^.

Moreover, the P2X_7_ receptor has been demonstrated to be mainly responsible for ATP-induced macrophage death^20^. The cell death of macrophages occurred only upon exposure to high concentration of exogenous ATP (2–5 mM, as shown in Supplementary Fig. S1) . Such high agonist concentration will not only open cation channels, but also lead to the formation of large cytotoxic transmembrane pores^13^,^14^. The formation of permeable pores disrupts ionic gradients and induces an efflux of vital intracellular molecules of maximum 900 Da, which may lead to cell death^13^,^14^,^15^. Although how P2X_7_ receptor activation causes cell death is not fully understood, some reports suggested that the caspases activation depending on mitochondrial pathway was involved in the P2X_7_ receptor-mediated apoptosis^16^. The present data indicated that stimulation with high extracellular ATP (5 mM) resulted in impairment of mitochondria, activation of caspase 3/7 (Figs 7 and 8), and eventually initiation of apoptotic cell death in rat peritoneal macrophages. Besides, our experiments with P2X_7_ receptor selective antagonist BBG (Figs 2, 3, 4, 5, 6, 7, 8) and irreversible antagonist oxidized ATP (Supplementary Fig. S2) confirmed the contribution of P2X_7_ receptors to these ATP-induced responses.

The most significant finding of present study was that we discovered and demonstrated that a natural anthraquinone derivative, rhein, could consistently inhibit all the P2X_7_ receptor-mediated responses examined here. Firstly, it strongly suppressed ATP-elicited increases of [Ca^2+^]_c_ and formation of the EB dye permeable pore in both rP2X_7_-HEK293 cells (Fig. 1) and rat peritoneal macrophages (Figs 2 and 3), in a concentration dependent manner, respectively. Secondly, it effectively prevented ATP/BzATP-evoked intracellular phagocytosis attenuation (Fig. 4), ROS production (Fig. 5), and massive secretion of IL-1β (Fig. 6) in rat peritoneal macrophages in a concentration dependent fashion. Rhein also reduced macrophage death caused by prolonged ATP stimulation (Fig. 7). Moreover, rhein potently reversed ATP-induced reduction in MMP and suppressed ATP-triggered caspases activation (Fig. 8). These results taken together provided compelling evidence to show the potent inhibitory effects of rhein on P2X_7_ receptor-related consequences.

Actually, nowadays more and more attentions are being paid to the anti-inflammatory activity of rhein. Clinical applications have also demonstrated that rhein may be a potential cure for inflammatory diseases. For instance, rhein is an active metabolite for the drug of osteoarthritis diacetylrhein. Although the underlying mechanisms of the anti-inflammatory effect of rhein remain to be elucidated, studies so far have focused on the effects of rhein on the factors known to be critical in the pathogenesis of osteoarthritis^39^. It has been reported that rhein could significantly inhibit LPS-induced production of IL-1β and IL-1 downstream signaling events such as nuclear factor-κB activation and nitric oxide generation^36^,^37^. Rhein could also block superoxide anion production, chemotaxis, phagocytosis and migration in neutrophils^46^ and macrophages^38^. As mentioned above, many of these processes are now known to depend on the P2X_7_ receptor activation. Thus, the P2X_7_ receptor serves as a good candidate target of rhein.

Importantly, we obtained all the IC_50_ values of rhein against rat P2X_7_ receptor-mediated responses, which were ranged from 0.4 to 1.75 μM. This is significantly lower than that of IC_50_ values of rhein in other characterized cellular effects. For instance, rhein inhibited IKKβ in LPS-activated macrophages with an IC_50_ of 11.79 μM^38^. Rhein blocked IL-1β-induced activation of MEK/ERK pathway in cultured chondrocytes with hypoxia^36^, and protected endothelial cell from oxidative stress at ∼10 μM magnitude^35^. Rhein-induced Hep-G2 cell death via mitochondrion permeability transition only occurred at concentrations higher than 100 μM^34^. Such reports indicated that rhein was a more potent antagonist against rat P2X_7_ receptors. Furthermore, the results from rP2X_7_-HEK293 cells suggested that rhein was highly specific and selective for P2X_7_ receptor rather than other P2X receptors. Since rhein is a natural constituent of traditional Chinese herb, the discovery of rhein represents a significant advance in P2X_7_ receptor pharmacology due to its significant anti-inflammatory activities. Besides, rhein showed nearly equivalent potency in blocking P2X_7_ receptor-mediated calcium increase and pore formation in both rP2X7-HEK293 cells and macrophages, whereas the rat selective antagonist BBG and the human selective antagonist KN-62 are more potent (>10-folds) in inhibiting the P2X_7_ receptor-mediated pore formation than blocking the receptor-mediated calcium influx^40^,^41^,^47^. What’s more, rhein also showed significant potency to block other P2X_7_ receptor-mediated important inflammatory processes including ROS generation, phagocytic activity and cell death of macrophages, which have rarely been investigated in other reports on the antagonists of P2X_7_ receptor.

In summary, our study clearly demonstrated that rhein could inhibit ATP/BzATP-induced various inflammatory responses including cytosolic calcium increase, membrane permeable pore formation, intracellular ROS production, IL-1β release, phagocytosis attenuation and cell apoptosis in rat peritoneal macrophages through antagonizing the P2X_7_ receptor. Our finding provides a novel insight into the molecular mechanism or pathway underlying the anti-inflammatory effects of rhein. Furthermore, because of the important roles of P2X_7_ receptor in immune response, inflammation and inflammatory diseases, the present investigation of rhein may facilitate development of therapeutic drugs targeting the P2X_7_ receptors.

## Methods

### Ethics Statement

All experimental protocols were approved by the Institutional Animal Care and Use Committee at Nankai University (Approval ID 201009080081). All experimental procedures involving animals and their care were carried out in accordance with the Institutional Animal Care and Use Committee at Nankai University and National Institutes of Health Guide for the Care and Use of Laboratory Animals.

### Animals and reagents

Healthy male Wistar rats with weight of 200 ± 50 g were obtained from Institute of Health and Environmental Medicine, Academy of Military Medical Sciences (Tianjin, China, Certification Number: SCXK 2010-0002). Dulbecco's modified Eagle's medium (DMEM), RPMI1640 medium and fetal calf serum (FCS) were purchased from Gibco (USA) and HyClone (USA), respectively. Rhein was obtained from National Institute for the Control of Pharmaceutical and Biological Products (China). Fura-2/AM was from Biotium (USA). Rat IL-1β ELISA kits were purchased from NeoBioscience Technology Co.,Ltd. (China). RNAprep pure Cell/Bacteria Kit was from TIANGEN BIOTECH (China). Reverse Transcription System, GoTaq PCR Core system and Caspase-Glo assay kit were from Promega (USA). FluoSpheres carboxylate-modified, 1.0 μm, nile red was purchased from Molecular Probes (USA). The rest of reagents, including ATP, BzATP, oxidized ATP, BBG, LPS, EB, DHE, rhodamine 123, dimethylsulfoxide (DMSO) and MTT were purchased from Sigma (USA).

### Cell preparation and culture

Wistar rats were sacrificed according to institutional guidelines. Then, Hanks’ balanced salt solution (HBSS) (NaCl 150 mM, KCl 5.4 mM, CaCl_2_ 2 mM, MgCl_2_ 1 mM, glucose 10 mM and HEPES 10 mM, pH = 7.4) was injected into the abdomen of each rat. Peritoneal cells were collected from the abdomen and isolated by centrifugation at 200 *g* for 10 min, which were subsequently cultured in RPMI1640 medium containing 10% FCS in a humidified incubator with 5% CO_2_ at 37 °C before analysis. The adherent cells were used in our experiment. Nonspecific esterase staining showed that approximately 95% of them were macrophages as previously described^44^. The rP2X_7_-HEK293 cells, a generous gift from Dr. Lin-Hua Jiang (Institute of Membrane and Systems Biology, Faculty of Biological Sciences, University of Leeds, Leeds, UK), were maintained in DMEM supplemented with 10% FCS and 2 mM L-glutamine in a humidified 5% CO_2_ incubator at 37 °C.

### RNA isolation and RT-PCR

Total RNA from rat peritoneal macrophages was isolated using RNAprep pure Cell/Bacteria Kit. Then, the RNA (1 μg) was subjected to reverse transcription (RT) using a RT system (Promega, USA) in a total volume of 20 μl reaction that contained MgCl_2_ (5 mM), dNTP mixture (1 mM), oligo(dT)_15_ primer (0.5 μg), RNase inhibitor (0.5 μl), reverse transcription 10 × buffer (2 μl), and AMV reverse transcriptase (15 U). The reaction mixtures were incubated at 45 °C for 30 min, 99 °C for 5 min to inactivate the enzyme, and then chilled on ice for 5 min. Subsequently, the product of RT reaction (1 μl) was amplified using a Go Taq PCR Core system (Promega, USA) in a total volume of 50 μl PCR buffer containing Green Master Mix (25 μl), sense primer (100 pM) and antisense primer (100 pM). The reaction mixtures were preheated to 95 °C for 2 min followed by 40 thermal cycles in a PCR machine (MJMini^TM^, BIO-RAD, USA). For each cycle, denaturation was at 95 °C for 30 s, annealing at 63.5 °C for 30 s, and extension at 72 °C for 1 min. PCR primers were as follows: P2X_7_ sense: 5′-CCCTGGCTACAACTTCAGATACGC-3′, antisense: 5′-GCTCCACGATGGGCTCACAC-3′, encompassing 317 bp of the published rat P2X_7_ sequence; GAPDH (glyceraldehyde-3-phosphate dehydrogenase, as positive control) sense: 5′-GTGGAGTCTACTGGCGTCTT-3′, antisense: 5′-CCAGGATGCCCTTTAGTG - 3′, encompassing 537 bp.

### Measurement of [Ca^2+^]_c_ in single cells

[Ca^2+^]_c_ was measured as previously described^45^. In brief, macrophages or rP2X_7_-HEK293 cells were loaded with 2 μM fura 2-AM in HBSS for 1 h, followed by extensive washing. Then, cells were bathed in fresh HBSS solution for monitoring the changes of [Ca^2+^]_c_. [Ca^2+^]_c_ was measured with calcium imaging system built on an inverted fluorescence microscope (Olympus IX51). As a ratiometric fluorescent Ca^2+^ indicator, fura-2 was alternately excited at 340 nm and 380 nm with a Lambda 10–2 Sutter (Sutter Instrument, USA). Fluorescence images (filtered at 515 nm ± 25 nm) were captured by a CCD camera (CoolSNAP fx-M, Roper Scientific, USA) and analyzed with MetaFluor software (Universal Imaging, PA, USA). [Ca^2+^]_c_ was represented by the ratio of fluorescence intensity at 340 nm/fluorescence intensity at 380 nm (F340/F380). At least three independent experiments were done for each condition. One curve of calcium changes was plotted as the representation of other similar traces.

### EB Dye uptake assay

Macrophages or rP2X_7_-HEK293 cells were seeded in 6-well plates at ∼1 × 10^6^/well and incubated for 4 h. Then, cells were pretreated with rhein or BBG at indicated concentrations for 20 min. Subsequently, ATP was added for 10 min in the presence of 6 μM EB. After extensive washing with HBSS solution, dye uptake positive cells were identified by illuminating with 488 nm light and detecting emission at 610 nm using an Olympus fluorescence microscope with a 20 × objective. The fluorescent intensity that indicates EB uptake in each cell was measured individually using MetaFluor software. The summation of intensity was divided by cell number to get the average intensity (F). The EB uptake in ATP alone group (F_ATP_ - F_control_) was taken as 100% (control group was treated with vehicle solution), and the percentages of EB uptake in other groups were normalized to the ATP group and calculated as: (F_test_–F_control_)/(F_ATP_–F_control_) × 100%.

### Phagocytosis of fluorescent microspheres assay

The phagocytic activity of macrophages was assessed by the uptake of 1.0 μm diameter nile red fluorescent carboxylate-modified microspheres. After pretreatment with different concentrations of rhein or BBG for 3 h in serum-free DMEM medium at 37 °C, ATP (5 mM) or BzATP (0.5 mM) and microspheres (10 μl) were applied to macrophages for 45 min, then washed thoroughly with ice cold PBS. Samples were quenched with PBS 1% BSA. The number of fluorescent particles taken up per macrophage was summarized to evaluate the phagocytic ability. The data were normalized to phagocytosis obtained on untreated macrophages. For imaging analysis, microphages were prepared in poly-L-lysine-coated glass coverslips at a density of 1 × 10^6^ cells/ml. The images were captured by a Zeiss fluorescent microscope (Axio observer D1) with a 100 × oil immersion lens.

### Measurement of IL-1β release

Extracellular IL-1β was detected by the rat IL-1β ELISA kit. Briefly, after treatment with indicated reagents, the supernatant of macrophages in culture in each group was transferred into ELISA plate seeded with the IL-1β antibody, and incubated for 90 min. Then the plate was washed with the washing buffer for five times. After that, the secondary antibody was applied for 60 min and then washed extensively. Subsequently, the HRP-Conjugate reagent was introduced to each well of the plate to promote the formation of antibody-antigen-enzyme-antibody compounds for 30 min. After repeated washing steps, the chromogen solution and stop solution was sequentially added into each well for an incubation step of 15 min and 5 min, respectively. Finally, the absorbance at 450 nm representing the relative level of IL-1β in each well was measured by an ELISA reader (Bio-Rad Imark). The concentration of IL-1β was determined by the standard curve.

### Detection of intracellular ROS

DHE, a reduced form of ethidium bromide, was used to determine intracellular ROS. After treatment with indicated reagents, macrophages were incubated in HBSS with 5 μM DHE for 30 min at 37 °C in dark. Then they were rinsed twice with HBSS and observed by a fluorescence microscope at the excitation wavelength of 488 nm and emission wavelength of 610 nm. The fluorescent intensity represents the intracellular ROS level.

### Cell death assay

Macrophages were seeded in 96-well plates at ∼1 × 10^5^ cells/well and cultured for 24 h. After pretreatment with rhein or BBG for 2.5 h, application of ATP (5 mM) to macrophages for 30 min. Then, cells were incubated in fresh culture medium for a further 24 h. Upon addition of 10 μl MTT reagents (10 mg/ml in HBSS) into each well for 4 h, the culture medium was aspirated and replaced with 100 μl lysis solution (50% DMSO and 50% ethanol). The absorbance at 570 nm (A_570_) for each well was determined by an ELISA reader. The percentage of survival cells was derived as: A_570, test_/A_570, control_ × 100%, and the percentage of dead cells was calculated as: (A_570, control_ – A_570, test_)/A_570, control_ × 100%.

### Detection of mitochondrial membrane potential

Rhodamine 123 was used to assess the depolarization of mitochondrial membrane potential (MMP). Macrophages were seeded into 96-well plate at ∼1 × 10^5^ cells/well and incubated in HBSS with rhodamine 123 (10 μg/ml) for 15 min at room temperature after treating with or without test agents. The rhodamine 123-loaded cells were washed and imaged with an inverted fluorescence microscope. The excitation wavelength and emission wavelength were 488 nm and 510 nm, respectively. A decrease in rhodamine 123 fluorescence intensity represents mitochondrial membrane depolarization.

### Caspase assay

Caspase 3/7 activities in macrophages were measured to determine the apoptotic events, using a Caspase-Glo assay kit. Briefly, the luminogenic substrate containing the tetrapeptide sequence DEVD is cleaved by caspase 3/7. After caspase cleavage, a luciferase substrate is released, resulting in the luciferase reaction and the production of luminescent signal. Cell suspension (200 μl; 1 × 10^5^ cells/ml) was seeded into a 96-well plate and incubated with or without test reagents at 37 °C in DMEM with 5% FBS. After that, an equal volume of reagents was added to each well. Then, samples were incubated at room temperature for 2 h. Finally, the luminescence of each sample was measured by a luminometer (GloMax Multi Jr Detection System, Promega, USA). These fluorescent data from each group were proportional to the amount of caspase activity, which was assigned as the relative fluorescence unit (RFU).

### Data analysis

All data were presented as mean ± standard deviation (SD). The statistical comparison between two groups was carried out using Student’s *t*-test (Origin 8.5), and the analysis for multiple groups was using Dunnett's test (SPSS 18.0, one-way ANOVA). *P* < 0.05 was considered to be statistically significant. The concentrations producing half of the maximal inhibition (IC_50_) were derived by fitting of the mean data to the Boltzmann equation: 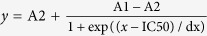, in which *y* is the inhibition ratio of increase in [Ca^2+^]_c_, *A1* is the asymptotic maximum, *A2* is the asymptotic minimum, *x* is concentrations of rhein and *dx* is the time constant.

## Additional Information

**How to cite this article**: Hu, F. *et al.* Rhein antagonizes P2X_7_ receptor in rat peritoneal macrophages. *Sci. Rep.* **5**, 14012; doi: 10.1038/srep14012 (2015).

## Supplementary Material

Supplementary Information
