# Supplementary material for: Rhein antagonizes P2X7 receptor in rat peritoneal macrophages
Source: Sci Rep. 2015 Sep 10;5:14012. doi: 10.1038/srep14012 (PMC4564849; doi:10.1038/srep14012)

**Rhein antagonizes P2X_7_ receptor in rat peritoneal macrophages**

Fen Hu^1#^, Fulin Xing^1#^, Ge Zhu^1^, Guangxue Xu^2^, Cunbo Li^1^, Junle Qu^3^, Imshik Lee^1^, Leiting Pan^1,4^*

*^1^The Key Laboratory of Weak-Light Nonlinear Photonics of Education Ministry, School of Physics and TEDA Applied Physics Institute, Nankai University, Tianjin, China*

*^2^School of Life Sciences, Lanzhou University, Lanzhou, China*

^3^*Shenzhen Key Laboratory of Micro-Nano Measuring and Imaging in Biomedical Optics, College of Optoelectronic Engineering, Shenzhen University, Shenzhen, China*

*^4^State Key Laboratory of Medicinal Chemical Biology, Nankai University, Tianjin, China*

**Figure S1 The effects of different concentrations of ATP on [Ca^2+^]_c_, pore formation and cell viability.** (a) Representative tracings of F340/F380 ratio for cells treated with different concentrations of ATP at 1 μM, 10 μM, 100 μM, 1 mM, 2 mM, 5 mM, respectively. (b) Summary of increase in F340/F380 for cells treated with different concentrations of ATP alone (black) or together with 1 μM rhein (green) (*n* =20 cells for each case). Arrows indicated applications of ATP. *, *P* < 0.05, compared to each dose of ATP alone group. (c) Representative phase contrast images visualizing all cells (upper panel) and ethidium bromide fluorescent images showing dye uptake positive cells (lower panel). Cells were pretreated with different concentrations of ATP at 0, 10 μM, 100 μM, 1 mM, 2 mM, 5 mM, respectively. (d) Summary of the fluorescence intensity measured in each experimental group (*n* = 150 cells for each case). *, *P* < 0.05, compared to control group. (e) Summary of cell death percentage in each group treated with different concentrations of ATP at 0, 100 μM, 1 mM, 2 mM, 5 mM, respectively. (*n* = 6). *, *P* < 0.05, compared to control group.

**
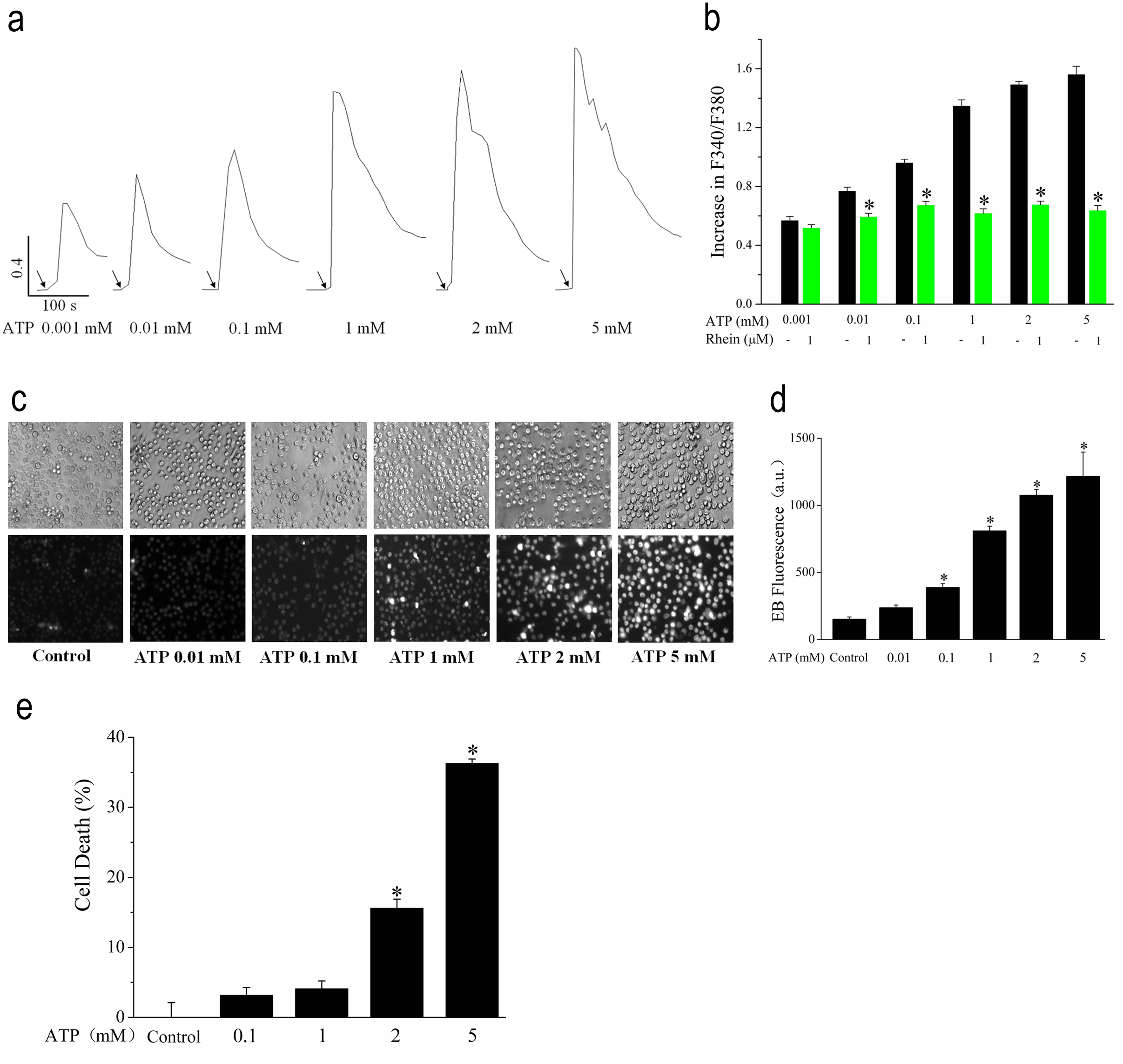
**

**Figure S2 OxATP inhibited ATP-induced [Ca^2+^]_c_ increase and pore formation in rat peritoneal macrophages.** (a) Representative tracings of F340/F380 ratio for cells treated with ATP (5 mM)/BzATP (0.5 mM) or together with OxATP (500 μM). Arrows indicated applications of ATP or BzATP. (b) Summary of increase in F340/F380 measured in each experimental group (*n* =20 cells for each case) *, *P* < 0.05, compared to ATP alone group. #, *P* < 0.05, compared to BzATP alone group. (c) The cells were pretreated with OxATP (500 μM) for 3 h, then, stimulated with ATP (5 mM) or BzATP (0.5 mM) for additional 30 min. (d) Summary of the fluorescence intensity measured in each experimental group (*n* = 150 cells for each case). *, *P* < 0.05, compared to control group. #, *P* < 0.05, compared to ATP alone group. &, *P* < 0.05, compared to BzATP alone group.


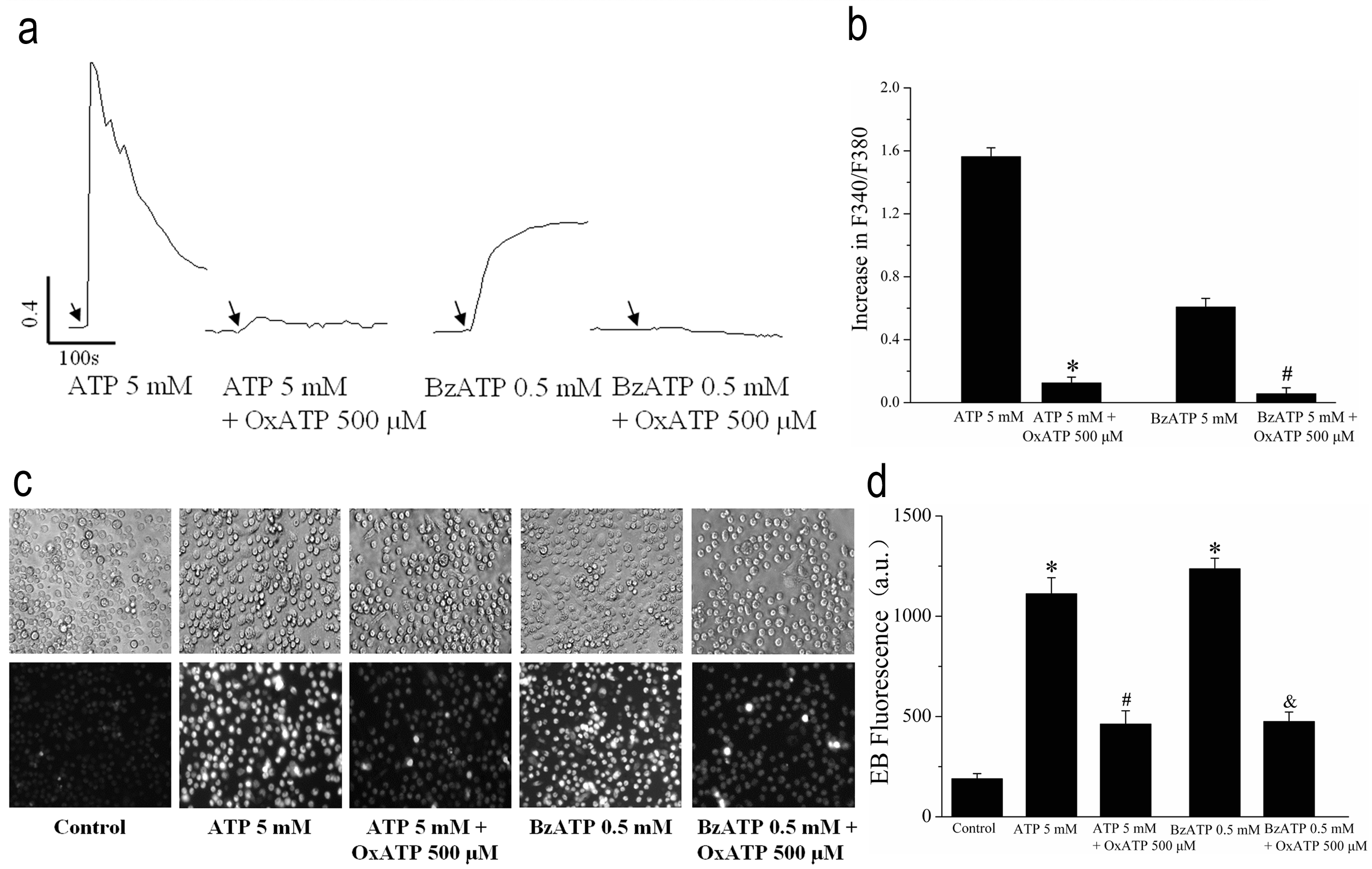

Supplement: Supplementary Information [file srep14012-s1.doc]
